# Interoceptive Insular Cortex Mediates Both Innate Fear and Contextual Threat Conditioning to Predator Odor

**DOI:** 10.3389/fnbeh.2019.00283

**Published:** 2020-01-09

**Authors:** María Rodríguez, Francisco Ceric, Paola Murgas, Bruce Harland, Fernando Torrealba, Marco Contreras

**Affiliations:** ^1^Departamento de Fisiología, Facultad de Ciencias Biológicas, Pontificia Universidad Católica de Chile, Santiago, Chile; ^2^Laboratorio de Neurociencia Afectiva, Facultad de Psicología, Universidad del Desarrollo, Santiago, Chile; ^3^Centro de Biología Integrativa, Facultad de Ciencias, Universidad Mayor, Santiago, Chile; ^4^Department of Psychology, University of Arizona, Tucson, AZ, United States

**Keywords:** insular cortex, threat response, defensive behavior, learned fear, cat odor, freezing

## Abstract

The insular cortex (IC), among other brain regions, becomes active when humans experience fear or anxiety. However, few experimental studies in rats have implicated the IC in threat responses. We have recently reported that inactivation of the primary interoceptive cortex (pIC) during pre-training, or the intra-pIC blockade of protein synthesis immediately after training, impaired the consolidation of auditory fear conditioning. The present study was designed to investigate the role of the pIC in innate and learned defensive responses to predator odor. Freezing behavior was elicited by single or repetitive exposures to a collar that had been worn by a domestic cat. Sessions were video-recorded and later scored by video observation. We found that muscimol inactivation of the pIC reduced the expression of freezing reaction in response to a single or repeated exposure to cat odor. We also found that pIC inactivation with muscimol impaired conditioning of fear to the context in which rats were exposed to cat odor. Furthermore, neosaxitoxin inactivation of the pIC resulted in a prolonged and robust reduction in freezing response in subsequent re-exposures to cat odor. In addition, freezing behavior significantly correlated with the neural activity of the IC. The present results suggest that the IC is involved in the expression of both innate and learned fear responses to predator odor.

## Introduction

The insular cortex (IC) is involved in the processing of visceral (also referred to as interoceptive; Cechetto and Saper, 1987; Hanamori, 2005) and emotional (Damasio et al., 2000) signals. In the rat, the primary interoceptive cortex (pIC) is located in the posterior granular insular cortex (Contreras et al., 2007) and receives visceral inputs from diverse interoceptive receptors via the thalamus and the parabrachial nucleus (Cechetto and Saper, 1987; Allen et al., 1991). The pIC is reciprocally connected with the anterior insula, the higher-order association region of the IC, which in turn sends projections to the prefrontal cortices and amygdala, among other brain areas (Shi and Cassell, 1998; Saper, 2002). Interestingly, a similar connectivity pattern has been reported in monkeys (Mesulam and Mufson, 1985) and humans (Critchley and Harrison, 2013) indicating high evolutionary conservation of the interoceptive system in mammals.

Life-threatening situations elicit defensive behavioral responses which increase the likelihood of an organism’s survival since these behaviors are effective in avoiding or reducing harm in dangerous situations such as exposure to a predator (Blanchard et al., 2001; Gross and Canteras, 2012). Defensive behaviors are seen in different animal species and are rapidly associated to stimuli and situations related to threats.

Antipredator defensive behavior patterns have been extensively studied in rodents. For instance, the stimulation of the olfactory and vomeronasal systems by a predator cue-elicited vigorous defensive responses (Papes et al., 2010), including freezing and risk assessment, which were accompanied by changes in endocrine and neural activity (Blanchard et al., 1998; Dielenberg and McGregor, 2001). Studies in rats have shown that freezing behavior is the dominant defensive response to an inescapable threat situation whereas risk assessment prevails when the threat is ambiguous or non-localized (Blanchard and Blanchard, 2008).

The IC is connected to key structures involved in the elaboration of defensive behaviors, including the medial amygdala, the medial prefrontal cortex, the ventromedial hypothalamus, and midbrain sites such as the periaqueductal gray (PAG; Canteras et al., 1994, 1995; Shi and Cassell, 1998). It remains unclear, however, whether the IC is involved in the expression of innate and learned defensive responses to predator odor.

Only a few studies have explored the role of the IC in learned fear in rodents. We have recently reported that pre-training inactivation of the pIC or the intra-pIC blockade of protein synthesis immediately after training impaired the consolidation of auditory fear conditioning (Casanova et al., 2016). These results are consistent with previous findings showing that the inactivation of the anterior IC immediately after training attenuated the behavioral and cardiovascular responses to the training context, suggesting the involvement of the IC in the consolidation of contextual fear memory (Alves et al., 2013).

Moreover, it has also been shown that fear and anxiety responses may be modulated by visceral afferent signals. Rats with subdiaphragmatic vagal deafferentation showed reduced anxiety levels and attenuation of conditioned fear extinction (Klarer et al., 2014). Furthermore, in humans, anxiety disorders have been associated with altered interoceptive processing in the insular cortex (Paulus and Stein, 2010; Simmons et al., 2011).

In this study, we assessed the effects of short-lasting (muscimol) or long-lasting (neosaxitoxin, NSTX; Casanova et al., 2016) pIC inactivation on the expression of innate and learned fear in rats exposed to cat odor. We further examined whether cat odor-related neural activity in the IC was correlated with freezing response. We found that the IC is involved in the expression of both innate and contextual fear of predator odor.

## Materials and Methods

### Subjects

Sixty-eight adult male Sprague–Dawley rats weighing 270–290 g at the beginning of the procedures, were individually housed and kept in an inverted 12/12 h light/dark regime (lights on at 7:00 P.M). Animal procedures were conducted in compliance with institutional guidelines by the National Institute of Health (USA) Guide for the Care and Use of Laboratory Animals (NIH Publication No. 80–23, revised 1996) and approved by the institutional Bio-Safety and Ethical Committee.

### Surgical Procedures

Rats were anesthetized with 100 mg/Kg of ketamine (Imalgene, Rhodia Merieux) plus 20 mg/Kg of xylazine (Rompun, Bayer), and placed in a stereotaxic apparatus (Kopf, CA, USA) to implant sterile stainless-steel guide cannulae. Guide cannulae (26 gauge, Plastics One, Roanoke, VA, USA) were anchored to the skull with screws (Plastics One, Roanoke, VA, USA) and dental acrylic; an occluder sealed each guide cannula. The guide cannulae were targeted bilaterally at the following coordinates according to Swanson’s atlas (Swanson, 1998). Primary interceptive cortex (VISC, in Swanson’s nomenclature), Bregma −0.51 mm, midline 5.0 mm, depth from the cranial surface 4.5 mm; angled 10° medially from the vertical. The injection cannula (33 gauge, from Plastics One, Roanoke, VA, USA) protruded 2 mm beyond the tip of the guide cannula. The placement of the injection was verified by analyzing the location of the tip of the cannulae in Nissl stained sections. Antibiotics were administered at the end of the surgery (enrofloxacin 5%; 19 mg/kg i.p., Bayer) together with a single dose of the anti-inflammatory ketoprofen 1% (ketophen 0.2 mg/kg i.p., Rhodia Merieux). Rats were allowed to recover for 1 week before beginning behavioral testing.

### Cortical Injections

The injection cannula was coupled to a 1 μl Hamilton Syringe, filled with the GABA agonist muscimol (0.5 μg/μl, Sigma-Aldrich) or sterile saline and inserted into the guide cannula after removing the occluder. Injections were conducted slowly in a quiet awake animal to minimize jitter of the injection needle, and therefore possible diffusion. We injected 0.5 μl/hemisphere over 2 min, slowly removed the injection needle, and replaced the occluders immediately after the microinjection. For neosaxitoxin dihydrochloride (NSTX)—treated rats, the injection cannula was coupled to a 10 μl Hamilton Syringe by polyethylene tubing filled with this voltage-gated sodium channel blocker diluted in sterile saline (NSTX; 32.5 μM/μl/side, CRM-MRC Biotoxin, Canada) or filled with sterile saline. We injected 1 μl of NSTX or sterile saline during 2 min per hemisphere, slowly removed the injection needle, and immediately replaced the occluders. It has been shown that muscimol has a duration of action up to 9 h (Krupa et al., 1999) whereas NSTX completely abolished neural activity for 48 h (Galindo et al., 2018). A prolonged, but reversible, neural blockade by NSTX opens the possibility to assess the contribution of the IC to remote threat memory expression.

Our previous electrophysiological data showed that the neural activity was strongly suppressed up to a 1 mm radius after a 1 μl NSTX (32.5 μM) injection in the somatosensory cortex (Galindo et al., 2018). Histological analysis has also shown that the spread of 0.5–1 μl of muscimol infusions in the cortex is around 1 mm (Martin, 1991; Allen et al., 2008). We have previously successfully performed such muscimol and neosaxitoxin injections in the pIC and somatosensory cortex (same volume and injection parameters; Casanova et al., 2016). Considering the coordinates of the targeted injection site for the pIC used in the present work, the infusions of muscimol and NSTX are expected to be restricted to the posterior insula with minimal spread into the somatosensory cortex. Although NSTX blocks fibers of passage, the radial disposition of axons coming in and out of the cerebral cortex (Ramón y Cajal et al., 1995) makes this issue less relevant in the pIC.

### Cat Odor Test Procedures

#### Collar

The vinyl cat collars had a felt-lined inner face to better contain the cat odor (dimensions: width 1.5, thickness 0.5, length 30 cm). Testing collars were worn by a domestic female cat for a week and others were used as control collars (no odor). The cat was normally an indoor-outdoor animal and fed with regular commercial cat food, but was kept indoors while wearing the testing collars. The worn cat collars were kept in air-tight plastic containers and stored at 4°C. Every 3 days the testing collar was replaced by a collar containing fresh cat odor.

#### Cat Odor Test

We used a transparent Plexiglas rectangular chamber (60 × 40 × 40 cm, L, W, H) for testing. The behavioral sessions lasted 20 min, and rats were only exposed to the testing collar for the last 10 min of each session. Rats were habituated to the test chamber for 30 min per day over 3 days in the presence of the same unworn cat collar (familiar collar) before testing. During each test session, the animals were exposed to the familiar collar for 10 min and then depending on the experimental condition, the familiar collar was replaced with either a testing collar containing cat odor or a control collar (no cat odor) for an additional 10 min. All collars were fixed to the floor in the bottom right corner of the chamber.

At the end of each session, the chamber was cleaned with ethanol solution (5% v/v). All experiments were performed in a room illuminated by a red light bulb (80 watts) located 20 cm above the chamber. Video-recordings of the animal behavior were captured using a horizontally mounted video camera, and the behavioral analysis (see “Experimental Design” section) was scored off-line by an experimenter blind to the treatment groups.

### Histology

After completing the experiments the animals were deeply anesthetized with 7% chloral hydrate (350 mg/kg; i.p.) and perfused through the left ventricle with a saline flush (100 ml) followed by 500 ml of 4% paraformaldehyde in phosphate-buffered saline (PBS, pH 7.4). The brains were post-fixed in the same fixative for 2 h, then transferred to 30% sucrose with 0.02% sodium azide in PBS until they sank. Brains were cut frozen under dry ice in the coronal plane at 50 μm thickness using a sliding microtome. We obtained three alternating series of sections from each brain. For brain sections obtained from cannulated animals, one series was stained with cresyl violet to locate the tips of injection cannulae, and the other two series were stored in PBS at −20°C. For brain sections obtained from non-cannulated rats, one series was stained with cresyl violet and the other two were used for immunohistochemistry.

### Immunohistochemistry

Free-floating sections were incubated in 0.3% H_2_O_2_ in PBS for 30 min, rinsed in PBS and transferred to the blocking (0.4% Triton-X100, 0.02% sodium azide, 3% normal goat serum in PBS) solution for 1 h. The sections were then transferred to the primary antibody incubation solution and left there overnight at room temperature. This incubation solution contained the Fos antibody (rabbit polyclonal F7799, from Sigma, St. Louis, MO, USA) diluted 1:20,000 in the blocking solution. The sections were rinsed in PBS for 1 h before being incubated in the secondary antibody solution (Biotin-SP- conjugated AffiniPure goat anti-rabbit IgG (H + L) from Jackson ImmunoResearch, West Grove, PA, USA; diluted 1:1,000 in 0.4% Triton X100, 1.5% normal goat serum in PBS). After rinsing for 40 min, the sections were incubated for 1 h in Vectastain ABC Elite kit (Vector Laboratories, Burlingame, CA, USA) diluted 1:500 in PBS, rinsed and incubated in a 0.05% 3-3′ diaminobenzidine hydrochloride (DAB) solution containing 0.003% H_2_O_2_, and 0.05% nickel chloride to get a dark blue reaction product. The specificity of the c-Fos antibody has been tested by the pre-adsorption test (Constandil et al., 1995).

### Cell Counting

The number of Fos immunoreactive (Fos-ir) neurons was determined in coronal sections using a camera lucida and a 10× objective. The size of the counting grid was related to the size of the selected area. For the rostral agranular IC (RAIC; Jasmin et al., 2004), from Bregma +4.85 to +3.60 we used a 0.25 × 1 mm counting grid, from Bregma +2.80 we used a 0.5 × 1.25 mm counting grid, and from Bregma +1.70 to +1.20 we used a 0.5 × 1 mm counting grid. For the pIC, from Bregma 0.95 to −0.26 we used a 0.25 × 1 mm counting grid, and from Bregma −0.51 to −2.45 mm we used a 0.5 × 1 mm counting grid. For the posteroventral subnucleus of the medial amygdala (MeApv), from Bregma −2.45, the dorsomedial subnucleus of the ventromedial hypothalamic nucleus (VMHdm), from Bregma −2.00 to −2.85 and the lateral amygdala (LA), from Bregma −2.45 to −3.90, we used a 2.42 × 1.94 mm counting grid.

### Experimental Design

Predator odor elicits defensive behaviors, which may reflect both fear and anxiety in rodents. Fear may be measured as flight, avoidance or freezing, and anxiety may be measured as stretch postures and vigilant scanning (defined as observatory, side-to-side head movements, without locomoting), broadly known as risk assessment (Blanchard et al., 2001; Dielenberg and McGregor, 2001; Apfelbach et al., 2005). Here, we used a test chamber that helped us to reduce the extensive behavioral repertoire and amplify the physiological response to cat odor (Dielenberg et al., 2004). The rats were unable to escape or hide from the cat odor, hence they exhibited robust freezing behavior in response to cat odor exposure. Freezing response was defined as a complete absence of movements, except for respiration (Blanchard et al., 2005). The experiments were designed to assess whether the pIC is involved in innate defensive response, as well as, in recent and/or remote threat memory recall.

#### Experiment 1: Effect of Short-Lasting pIC Inactivation on the Expression of an Innate Defensive Response to Cat Odor

Rats were injected bilaterally with muscimol (0.5 μg/μl 0.5 μl/side) or saline (0.5 μl/hemisphere) into the pIC, and 30 min later, they were placed in the test chamber for 20 min. In the first 10 min, the animals were exposed to the unworn familiar collar, and then over the next 10 min, they were exposed to a collar containing cat odor or to an unworn collar (no cat odor, control collar).

#### Experiment 2: Effect of Short-Lasting pIC Inactivation on the Expression of Recent Threat Memory

Rats were injected bilaterally with muscimol (0.5 μg/μl 0.5 μl/side) or saline (0.5 μl/hemisphere) into the pIC, and 30 min later they were placed in the test chamber for 20 min. In the first 10 min, the animals were exposed to the unworn familiar collar and then over the next 10 min, they were exposed to a collar containing cat odor (Test, day 0). The next day, the rats were re-injected with muscimol or saline into the pIC, and 30 min later they were re-exposed to the test chamber for 10 min containing the unworn familiar collar (Context, day 1). Over the next 10 min, the animals were re-exposed to the cat odor (ReTest, day 1).

#### Experiment 3: Effect of Long-Lasting pIC Inactivation on Remote Threat Memory Recall

Rats were placed in the test chamber for 20 min. In the first 10 min, the animals were exposed to the unworn familiar collar and then over the next 10 min, they were exposed to a collar containing cat odor (Day 0). Immediately after the 10 min of cat odor exposure, the animals were injected bilaterally with NSTX or sterile saline in the pIC under brief isoflurane (Baxter Healthcare Corporation) anesthesia (2–1% in oxygen at a flow rate of ~3.0 l/min; less than 15 min). Twenty-four hours after the injection (ReTest), rats were placed in the test chamber and re-exposed to the unworn familiar collar for 10 min and then to cat odor for an additional 10 min. This procedure was repeated on the following day (ReTest 2) and 4 days (ReTest 3), 14 days (ReTest 4), and 29 days (ReTest 5) after the single Day 0 injection. It has been reported that isoflurane anesthesia could produce cognitive impairments in humans and rodents. However, these impairments were observed with longer exposures (>2 h) than those used here (<15 min) and were also controlled for here using saline injections (Carr et al., 2011; Callaway et al., 2012).

#### Experiment 4: Fos Immunoreactivity in the IC in Rats Exposed to Cat Odor

We assessed Fos immunoreactivity in the pIC in response to a single or repetitive exposure to cat odor in two separate groups of rats. The first group was exposed to the unworn familiar collar for 10 min followed by cat odor exposure for another 10 min (single exposure, Day 0). The second group experienced the same Day 0 procedure as the first, which was then repeated the following day (repeated exposure, Day 1). A control group was exposed to the unworn familiar collar for 20 min. Animals were perfused 90 min after the end of their last exposure test, and their brains were removed for immunohistochemical processing.

#### Statistical Analysis

Data were analyzed blind to condition. All statistical tests were performed using SPSS software (Version 20.0, Chicago, IL, USA). All data are expressed as mean ± standard error (SEM). Comparisons between groups were made using the Kruskal–Wallis *H*-test with the Mann–Whitney *U*-test for pairwise comparisons. The intra-group comparisons over time were analyzed with the Friedman test followed by the Wilcoxon signed-rank test for pairwise comparisons. Spearman’s rank-order correlations were performed to examine the relationship between neuronal activity in the IC and freezing behavior. In all figures, significance levels were set to <0.05 (*) and <0.01 (**).

## Results

### Histological Verification of the Injection Sites

**Figure 1:**
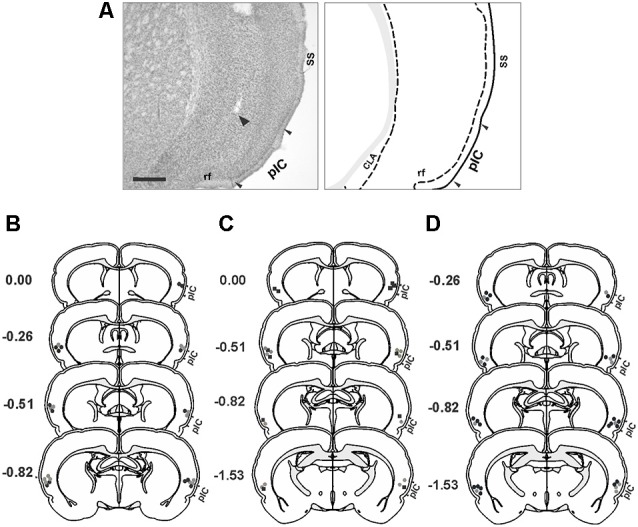
Histological analysis. **(A)** Photomicrograph of a Nissl-stained coronal section (left panel) showing the tip of the injection cannula track (arrowhead) in the primary interoceptive cortex (pIC) and a corresponding schematic drawing (right panel). **(B–D)** Reconstructions of the different injection sites of saline (gray circles), muscimol (black circles) and NSTX (black squares) into the pIC. **(B)** Experiment 1. **(C)** Experiment 2. **(D)** Experiment 3. Scale bar, 500 μm. Abbreviations: CLA, claustrum; rf, rhinal fissure; SS, somatosensory cortex.

Representative photomicrograph and schematics illustrating the injection cannula placements in the IC are shown in Figure 1. The histological analysis of the distribution of cannula placements in the insula revealed that the injection cannula tracks were located mainly in the posterior granular insular cortex, which contains the pIC in the rat (Contreras et al., 2007). Considering that the spread of neural inactivation following 0.5 μl of muscimol or 1 μl NSTX infusion is about 1 mm (Martin, 1991; Allen et al., 2008; Galindo et al., 2018), it is reasonable to conclude that adjacent areas such as the dysgranular area of the insular cortex, claustrum, and the secondary somatosensory cortex might have also been partially affected by the drugs.

### Experiment 1: Short-Lasting pIC Inactivation Impaired the Expression of Innate Defensive Freezing in Response to Cat Odor

**Figure 2:**
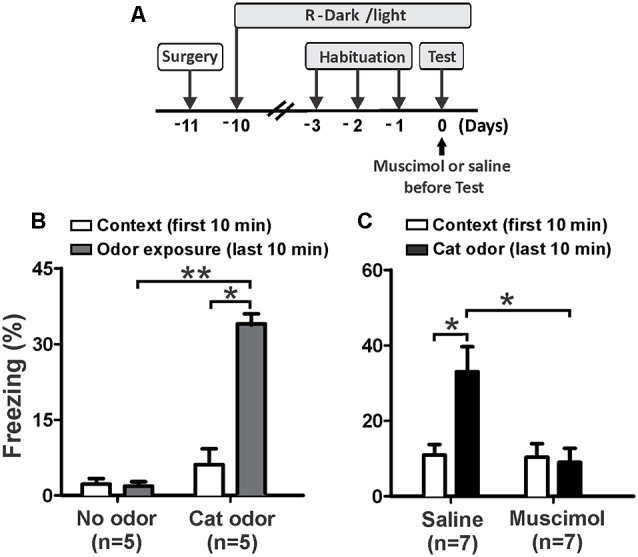
Inactivation of the pIC prior to the first exposure to cat odor abolished the innate freezing expression. **(A)** Timeline of the experimental design. **(B)** Rats were first exposed to a familiar control collar (Context) for 10 min and were then exposed either to a collar with or without cat odor for an additional period of 10 min (odor exposure). The bars show the percentage of time spent freezing displayed by non-implanted rats. **(C)** Percentage of time spent freezing displayed by implanted rats that received saline or muscimol into the pIC 30 min before testing and were exposed to cat odor. Data are expressed as means + SEM. *p < 0.05, **p < 0.01.

First, we investigated whether neural activity in the pIC is necessary for rodents to display innate defensive behavior. We decided to validate our behavioral assay using non-cannulated rats (cat odor and no odor groups) as control animals. We quantified freezing as a measure of antipredator defensive behavior. The rats exposed to a collar impregnated with cat odor showed a robust increase in freezing (Figure 2B, cat odor group, Wilcoxon Signed-ranks test, *Z* = −2.201, *p* = 0.028) compared with the unworn familiar collar (context exposure) in the first 10 min. Low level of freezing (Figure 2B, no odor group, Wilcoxon Signed-ranks test, *Z* = −0.184, *p* = 0.854) was observed in rats exposed to an unworn collar during the second part of the test. Further analysis revealed that the level of freezing (Figure 2B, Mann–Whitney test, *U* = 0.000, *p* = 0.004) was higher in the cat odor group than the no odor group during the second part of the test. There were no significant differences in freezing (Figure 2B, Mann–Whitney test, *U* = 11.000, *p* = 0.256) between the two groups during the first 10 min of exposure to the unworn familiar collar (context exposure). These results confirmed that this behavioral assay is suitable to study innate defensive behavior in rats.

We then assessed the effect of muscimol inactivation of the pIC on the expression of innate freezing behavior in response to cat odor (Figure 2A). Saline-infused rats showed a significant increase in freezing (Figure 2C, Wilcoxon Signed Ranks test, *Z* = −2.366, *p* = 0.018) during cat odor exposure. In contrast, rats infused with muscimol into the pIC showed comparable levels of freezing behavior during exposure to cat odor as they did with the unworn familiar collar (Figure 2C, Wilcoxon Signed Ranks test, *Z* = −0.530, *p* = 0.596). Additional analyses revealed that the level of freezing was higher in saline-infused rats than that seen in muscimol-infused animals during cat odor tests (Figure 2C, Mann–Whitney test, *U* = 5.500, *p* = 0.015). There were no significant differences in freezing (Figure 2C, Mann–Whitney test, *U* = 22.500, *p* = 0.798) between saline and muscimol infusions during the first 10 min of exposure to the unworn familiar collar.

These results indicate that muscimol inactivation of the pIC impaired the expression of unconditioned freezing in response to acute exposure to cat odor. Given that the IC has been implicated in contextual and auditory threat learning (Alves et al., 2013; Casanova et al., 2016), we then wanted to assess whether the pIC is involved in learned responses to cat odor.

### Experiment 2: Short-Lasting pIC Inactivation Impaired the Expression of Recent Threat Memory

**Figure 3:**
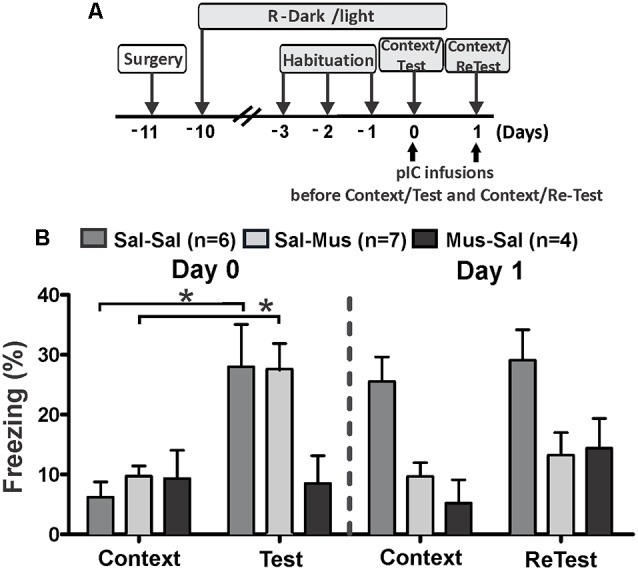
Muscimol inactivation of the pIC prevented contextual threat learning.** (A)** Timeline of the experimental design. Rats were first exposed to a control collar (Context) for 10 min and then exposed to cat odor (Test, ReTest) for an additional period of 10 min. Rats received saline or muscimol into the pIC 30 min before testing (Day 0). Next day (Day 1), rats that were injected with saline on Day 0 received a second saline or muscimol injection into the pIC 30 min before testing. Similarly, rats that received muscimol on day 0 were injected with saline into pIC 30 min before testing. **(B)** The bars show the percentage of time spent freezing. Data are expressed as means + SEM. *p < 0.05.

Next, we investigated the effect of short-lasting inactivation of the pIC on threat learning and recent memory recall using three experimental groups. On day 0, rats were injected with saline (Sal-Sal and Sal-Mus groups) or muscimol (Mus-Sal group) into the pIC 30 min prior to testing (Figure 3A). On the next day (day 1), rats that received saline on day 0 were injected with saline or muscimol in the pIC 30 min before testing. Similarly, rats injected with muscimol on day 0 were injected with saline into the pIC before testing on day 1.

Sal-Sal and Sal-Mus groups showed a significant increase in freezing (Figure 3B, Friedman Test, Sal-Sal, *H* = 12.800, *df* = 3, *p* = 0.003, Sal-Mus, *H* = 12.900, *df* = 3, *p* = 0.005) between testing days. Interestingly, the Mus-Sal group did not show a significant increase in freezing behavior (Figure 3B, Friedman Test, *H* = 4.469, *df* = 3, *p* = 0.215) between testing days.

*Post hoc* comparisons showed that during the first cat odor exposure (day 0, Test), Sal-Sal and Sal-Mus subjects showed significant increases in freezing (Wilcoxon Signed Ranks test, Sal-Sal, *Z* = −2.366, *p* = 0.018, Sal-Mus, *Z* = −2.521, *p* = 0.012) relative to the first context exposure (control collar). Consistent with our previous results (Experiment 1), the Mus-Sal group did not show a significant increase in freezing (Wilcoxon Signed Ranks test, *Z* = −1.355, *p* = 0.176) during the cat odor exposure compared to the context exposure on day 0.

The Sal-Sal group exhibited high levels of freezing (Wilcoxon Signed Ranks test, *Z* = −2.366, *p* = 0.018) in response to context exposure during day 1 relative to context exposure on day 0. There were no differences in freezing levels (Wilcoxon Signed Ranks test, *Z* = −1.1947, *p* = 0.051) between context and the second cat odor exposure (ReTest) on day 1 for the Sal-Sal group. Moreover, Sal-Sal rats exhibited the same levels of freezing (Wilcoxon Signed Ranks test, *Z* = −0.338, *p* = 0.735) during the second cat odor exposure (ReTest) compared to their first exposure (Test).

Sal-Mus subjects did not show differences in freezing (Wilcoxon Signed Ranks test, *Z* = −0.140, *p* = 0.889) levels between day 0 and day 1 context exposures. On day 1, no significant differences were found in freezing levels (Wilcoxon Signed Ranks test, *Z* = −0.840, *p* = 0.401) between context and the second cat odor exposure (ReTest) for Sal-Mus rats. Consistent with the result obtained in Experiment 1, freezing (Wilcoxon Signed Ranks test, *Z* = −2.100, *p* = 0.036) was significantly reduced in the Sal-Mus animals during ReTest compared with Test.

On day 1, the Mus-Sal group did not show an increase in freezing (Wilcoxon Signed Ranks test, *Z* = −1.214, *p* = 0.225) during the second cat odor exposure (ReTest) relative to context (control collar). Freezing levels (Wilcoxon Signed Ranks test, *Z* = −1.461, *p* = 0.144) did not differ significantly between the first and second context exposures in the Mus-Sal rats. Additionally, Mus-Sal rats did not show differences in freezing (Wilcoxon Signed Ranks test, *Z* = −0.944, *p* = 0.345) levels between Test and ReTest.

Additional analyses showed significant differences in freezing levels between groups during the first (Kruskal–Wallis Test, *H* = 7.105, *df* = 2, *p* = 0.0290) and second (Kruskal–Wallis Test, *H* = 7.159, *df* = 2, *p* = 0.028) cat odor exposures, and during the second (Kruskal–Wallis Test, *H* = 10.185, *df* = 2, *p* = 0.006), but not first (Kruskal–Wallis Test, *H* = 1.972, *df* = 2, *p* = 0.373) context exposures. *Post hoc* comparisons revealed that during cat odor exposure on day 0, Sal-Sal (Mann–Whitney test, *U* = 4.000, *p* = 0.028) and Sal-Mus (Mann–Whitney test, *U* = 3.000, *p* = 0.013) rats displayed higher levels of freezing than the Mus-Sal group. On day 1, Sal-Sal rats during ReTest displayed higher levels of freezing than the Sal-Mus group (Mann–Whitney test, *U* = 6.000, *p* = 0.011). Freezing levels did not differ between Sal-Sal rats and Mus-Sal rats (Mann–Whitney test, *U* = 6.000, *p* = 0.061).

These results strongly suggest that the neural activity of the pIC is not only important for displaying innate threat responses but also necessary for contextual threat conditioning.

### Experiment 3: Long-Lasting pIC Inactivation Impaired the Expression of Remote Threat Memory

**Figure 4:**
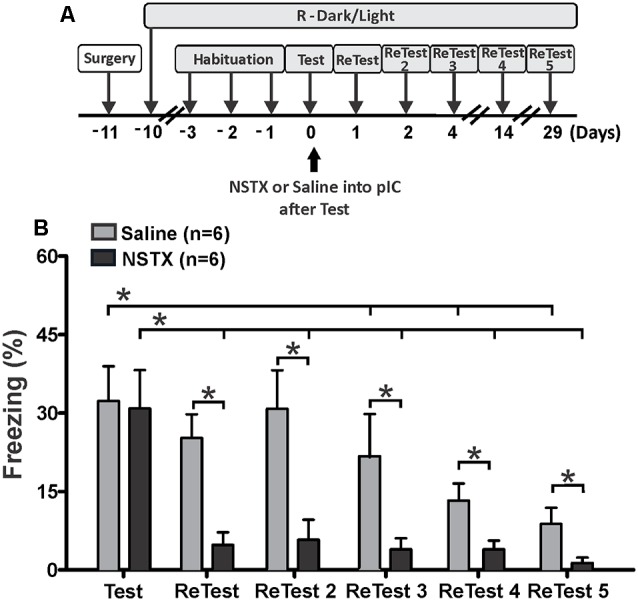
The pIC inactivation with neosaxitoxin (NSTX) caused a prolonged reduction in conditioned freezing response.** (A)** Timeline of the experimental design. Immediately after the first exposure to cat odor (Test), the rats were infused with saline or neosaxitoxin (NSTX) into the pIC. In the subsequent trials [ReTest (day 1), ReTest 2 (day 2), ReTest 3 (day 4), ReTest 4 (day14) and ReTest 5 (day 29)], they were re-exposed to cat odor. **(B)** The bars show the percentage of time spent freezing. Data are expressed as means + SEM. *p < 0.05.

We also explored the effect of long-lasting inactivation of the pIC on threat learning and remote memory recall. Rats received a single bilateral injection of NSTX or saline into the pIC immediately after the first 10 min exposure to cat odor (Figure 4A, Test). We re-exposed these animals to cat odor 24 h (ReTest), 48 h (ReTest 2), 4 days (ReTest 3), 14 days (ReTest 4) and 29 days (ReTest 5) after the injection. We previously reported that NSTX injections into the pIC did not produce motor impairments (Casanova et al., 2016) or tissue damage (Galindo et al., 2018).

The Friedman test showed a significant difference in freezing levels during cat odor exposures for NSTX-pIC (*H* = 18.134, *df* = 5, *p* = 0.003) and Sal-pIC (*H* = 26.555, *df* = 5, *p* = 0.000) groups. *Post hoc* comparisons confirmed that the NSTX inactivation of the pIC, but not saline infusion, resulted in a long-term reduction of freezing (Figure 4B). NSTX-pIC rats showed significantly reduced freezing levels during ReTest (Wilcoxon Signed Ranks test, *Z* = −2.366, *p* = 0.018), ReTest2 (Wilcoxon Signed Ranks test, *Z* = −2.366, *p* = 0.018), ReTest3 (Wilcoxon Signed Ranks test, *Z* = −2.366, *p* = 0.018), ReTest4 (Wilcoxon Signed Ranks test, *Z* = −2.197, *p* = 0.028) and ReTest5 (Wilcoxon Signed Ranks test, *Z* = −2.366, *p* = 0.018) relative to Test. Sal-pIC rats showed no differences in freezing levels during ReTest (Wilcoxon Signed Ranks test, *Z* = −1.897, *p* = 0.058) and ReTest2 (Wilcoxon Signed Ranks test, *Z* = −0.315, *p* = 0.752) relative to Test. However, we noted that after repeated exposure to cat odor, Sal-pIC rats showed lower levels of freezing during ReTest 3 (Wilcoxon Signed Ranks test, *Z* = −2.201, *p* = 0.028), ReTest4 (Wilcoxon Signed Ranks test, *Z* = −2.371, *p* = 0.018) and ReTest5 (Wilcoxon Signed Ranks test, *Z* = −2.366, *p* = 0.018) than during Test. Comparison between groups revealed that NSTX-pIC rats displayed significantly less freezing than Sal-pIC rats during ReTest (Mann–Whitney test, *U* = 1.500, *p* = 0.03), ReTest2 (Mann–Whitney test, *U* = 3.000, *p* = 0.06), ReTest3 (Mann–Whitney test, *U* = 8.000, *p* = 0.034), ReTest4 (Mann–Whitney test, *U* = 4.500, *p* = 0.010) and ReTest5 (Mann–Whitney test, *U* = 6.500, *p* = 0.018). No difference was observed during Test (Mann–Whitney test, *U* = 23.500, *p* = 0.898).

Overall, these results show that the long-lasting pIC inactivation induced a prolonged reduction of conditioned freezing in response to chronic cat odor exposure. These results confirm that the neural activity of the pIC is necessary for the expression of defensive behavior, and suggest that the pIC may be involved in the recall of remote fear memory.

### Experiment 4: Cat Odor Elicited Neuronal Activity in the Insular Cortex

**Figure 5:**
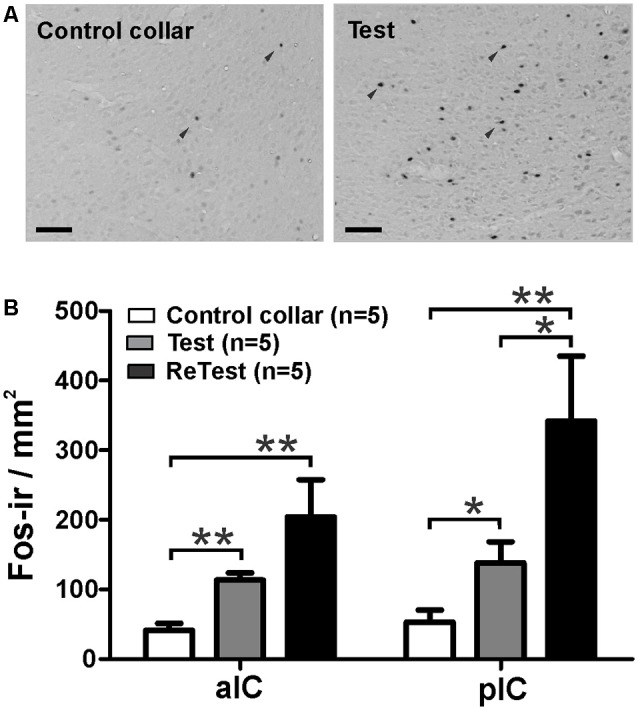
Cat odor exposure increased Fos expression in the insula cortex.** (A)** Representative photomicrograph of the pIC showing a near absence of Fos immunoreactive (Fos-ir) cells (black arrowheads) in rats exposed to a control collar (left) contrasted with a significant increase in the number of Fos-ir cells in rats exposed to cat odor (right). **(B)** Quantification of Fos-ir cells in the anterior insula cortex (aIC) and pIC in rats exposed once (Test) or twice (ReTest) to cat odor. Data are expressed as means + SEM. *p < 0.05, **p < 0.01. Scale bars, 200 μm.

Next, using Fos as a marker of neuronal activation (Chaudhuri and Zangenehpour, 2002), we assessed IC neural activity after cat odor exposure. Separate groups of animals were sacrificed 90 min after single or repeated exposures to cat odor (Figure 5). The Kruskal–Wallis test showed overall that there were significant differences in the neural activity of the pIC (*H* = 12.058, *df* = 2, *p* = 0.002) and the anterior insular cortex (aIC, *H* = 12.165, *df* = 2, *p* = 0.002) during cat odor exposures. *Post hoc* comparisons confirmed an elevated number of Fos-ir neurons in the pIC during the first (Test, Mann–Whitney test, *U* = 3.000, *p* = 0.016) and the second (ReTest, Mann–Whitney test, *U* = 0.000, *p* = 0.004) cat odor exposures when compared with the control collar group (Figure 5B). Remarkably, a greater number of Fos-ir neurons in the pIC were observed during ReTest (Mann–Whitney test, *U* = 2.000, *p* = 0.018) when compared with Test. Similarly, cat odor induced an increase in the number of Fos-ir neurons in the aIC during Test (Mann–Whitney test, *U* = 0.000, *p* = 0.002) and ReTest (Mann–Whitney test, *U* = 1.000, *p* = 0.004) when compared with the control collar. There was no significant difference in the number of Fos-ir neurons in the aIC between Test and ReTest (Mann–Whitney test, *U* = 6.000, *p* = 0.100).

**Figure 6:**
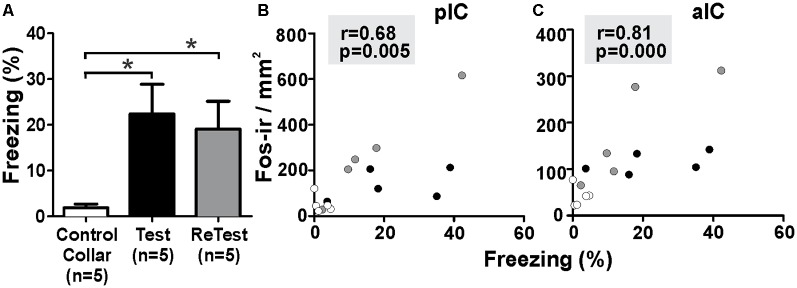
Fos expression in the insula cortex was highly correlated with freezing behavior. **(A)** Rats exposed to cat odor showed higher levels of freezing relative to the control collar condition during either the first (Test) or second exposure (ReTest). Data are expressed as means + SEM. *p < 0.05. **(B,C)** Scatter plots showing the relation between the percentage of time spent freezing and the number of Fos immunoreactive (Fos-ir) neurons in the posterior (pIC) and anterior (aIC) insula cortex for Test and ReTest. Coefficients and *p*-values are indicated.

To better understand the relationship between IC neural activity and the expression of defensive behavior, we conducted correlation analyses between freezing levels and the number of Fos-ir neurons in the IC during cat odor exposures (Figure 6). The Kruskal–Wallis test showed that overall there were significant differences in freezing levels during Test and ReTest (Figure 6A, *H* = 6.860, *df* = 2, *p* = 0.032). Further analysis showed high levels of freezing during Test (Mann–Whitney test, *U* = 2.000, *p* = 0.028) and ReTest (Mann–Whitney test, *U* = 2.000, *p* = 0.028) when compared to control collar with no difference in freezing between Test and ReTest (Mann–Whitney test, *U* = 9.000, *p* = 0.465). We also observed a high correlation between freezing levels and the neuronal activity of both the pIC (Figure 6B, Spearman’s Rank-Order *r* = 0.679 *p* = 0.005) and the aIC (Figure 6C, Spearman’s Rank-Order *r* = 0.807 *p* = 0.000). The analysis shows that the expression of freezing is positively correlated with an increase in IC neuronal activity.

**Figure 7:**
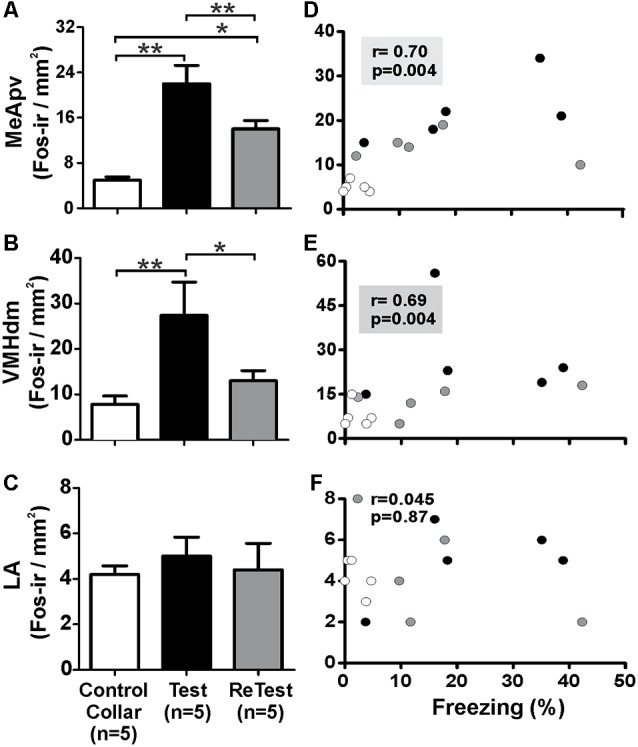
Fos expression in the MeApv and VMHdm correlated with freezing behavior. **(A–C)** Quantification of Fos immunoreactive (Fos-ir) cells in the posteroventral subnucleus of the medial amygdala (MeApv), dorsomedial subnucleus of the ventromedial hypothalamus (VMHdm) and lateral amygdala (LA), respectively. Data are expressed as means + SEM. *p < 0.05, **p < 0.01. **(D–F)** Scatter plots showing the relation between the percentage of time spent freezing and Fos-ir neurons in the MeApv, VMHdm, and LA, respectively. Coefficients and *p*-values are indicated.

To confirm that the cat odor was able to activate brain areas known to be involved in the innate fear circuit (Gross and Canteras, 2012; Silva et al., 2013), we measured Fos expression in the MeApvn and VMHdm. As shown in Figure 7, we found a significant increase in the number of Fos-ir neurons in both the MeApv (Figure 7A, Kruskal–Wallis Test, *H* = 14.296, *df* = 2, *p* = 0.001) and VMHdm (Figure 7B, Kruskal–Wallis Test, *H* = 11.266, *df* = 2, *p* = 0.004) during cat odor exposures (Test and ReTest), as expected from previous work (Blanchard et al., 2005; Silva et al., 2013). The Mann–Whitney test revealed that the number of Fos-ir neurons in the MeApv was higher during Test (*U* = 0.000, *p* = 0.004) and ReTest (*U* = 0.000, *p* = 0.004) than control collar. We also found a greater number of Fos-ir neurons in the MeApv during Tests relative to ReTest (Mann–Whitney test, *U* = 2.500, *p* = 0.013). We observed a correlation between a number of Fos-ir neurons in the MeApv and freezing levels (Figure 7D, Spearman’s Rank-Order *r* = 0.7 *p* = 0.04). Similarly, the proportion of Fos-ir VMHdm neurons was higher during Test (Mann–Whitney test, *U* = 0.500, *p* = 0.005), but not ReTest (Mann–Whitney test, *U* = 8.000, *p* = 0.106) when compared with control collar. We also found a greater number of Fos-ir neurons in the VMHdm during Test relative to ReTest (Mann–Whitney test, *U* = 2.000, *p* = 0.010). We observed a significant correlation between the proportion of Fos-ir VMHdm neurons and freezing levels (Figure 7E, Spearman’s Rank-Order *r* = 0.695 *p* = 0.004).

Additionally, we measured the Fos activity in the lateral nucleus of the amygdala (LA) which has been implicated in learned fear (Wallace and Rosen, 2001). We did not observe significant changes in Fos expression in the LA after a single or repeated cat odor exposure (Figure 7C, Kruskal–Wallis Test, *H* = 1.696, *df* = 2, *p* = 0.428). The proportion of Fos-ir neurons in the LA did not show a significant correlation with freezing levels (Figure 7F, Spearman’s Rank-Order *r* = 0.045 *p* = 0.872).

## Discussion

Our results show that the IC is involved in the expression of both innate and contextual threat responses to predator odor. We found that muscimol-induced inactivation of the pIC reduced the expression of freezing reaction to a single or repeated exposure to cat odor (Figures 2, 3). We also found that pIC inactivation with muscimol impaired context-dependent threat conditioning (Figure 3). Furthermore, long-lasting pIC inactivation (neosaxitoxin) resulted in a prolonged and robust reduction in freezing response in subsequent re-exposures to cat odor (Figure 4). We also observed that freezing behavior significantly correlated with the neural activity of the IC (Figure 6).

A growing body of evidence suggests that the interoceptive insular cortex plays an important role in learned threat responses (Berret et al., 2019). We have recently reported that inactivation of the pIC during pretraining, or the intra-pIC blockade of protein synthesis immediately after training, impaired the consolidation of auditory fear conditioning (Casanova et al., 2016). Here, we show that pIC inactivation reduced the expression of innate fear and impaired the contextual memory to predator odor in a similar way to the well-known effects of interfering with specific subcortical regions responsible for innate defensive behaviors (Blanchard et al., 2005).

Exposure to predator odor is a natural stressor for rodents that are known to activate the animal’s defensive system (Dielenberg and McGregor, 2001; Gross and Canteras, 2012). For instance, acute exposure of rats to cat odor induced a substantial increase in the neural activity of both MeApv and VMHdm, and increased defensive responses including freezing (Dielenberg et al., 2001b; Blanchard et al., 2005). Lesions of the MeA reduced freezing after exposure to predator odor (Blanchard et al., 2005) or to a live cat (Martinez et al., 2011), and the optogenetic stimulation of VMHdm generated autonomic and behavioral responses that resemble the animals’ natural defensive behaviors (Wang et al., 2015). Interestingly, the IC is connected to key structures of the defensive system, including the MeA, VMHdm, prefrontal cortex, and midbrain sites such as the PAG (Canteras et al., 1994, 1995; Shi and Cassell, 1998). Although it remains unclear whether the IC interacts with these regions for modulating innate defensive responses, a recent study showed that the IC acting through the amygdala modulates learned fear. The photoinhibition of neurons in the posterior IC that project to the lateral amygdala impaired the formation of auditory threat memory whereas the inhibition of IC neurons projecting to the central amygdala reduced freezing behavior during threat learning (Berret et al., 2019).

Functional studies indicate that the IC is important for representing the physiological state of the body, which may influence decision-making, motivation, memory, and emotional processes (Craig, 2002; Damasio and Carvalho, 2013; Garfinkel and Critchley, 2016). It is well known that numerous interoceptive changes take place during threat responses. For example, increments in heart rate, blood pressure (LeDoux et al., 1988; Garfinkel and Critchley, 2016) and plasma glucocorticoid levels (Figueiredo et al., 2003) have been observed in rats exposed to threat-related stimuli. These autonomic and endocrine changes (i.e., fear-related bodily states) may be represented in the interoceptive network, initially in the nucleus tractus solitarius (Claps and Torrealba, 1988; Torrealba and Claps, 1988), and then within the insular cortex. Interestingly, inactivation of the parabrachial nucleus, which is the predominant target of the nucleus tractus solitarius, impaired fear learning in mice (Sato et al., 2015). Studies in rats (Cechetto and Saper, 1987; Contreras et al., 2007, 2012) and humans (Craig, 2002; Critchley and Harrison, 2013) have suggested that the pIC maps the viscerosensory state, while the anterior insula, which is reciprocally interconnected with the pIC, is thought to be involved in the perception of visceral sensations and interoceptive memory. Thus, it seems reasonable to hypothesize that the sensory representation of fear-related bodily states in the pIC and subsequently within the anterior insula may modulate defensive responses to predator odor. Moreover, the IC is connected with the olfactory bulb (Shipley and Geinisman, 1984), the pyriform cortex (Gerfen and Clavier, 1979) and as was mentioned above, with structures of the defensive system such as the amygdala. Taken together, this suggests that the IC may be a key cortical site where olfactory and fear-related visceral information is integrated, and encoded, into long-term memory. A similar idea has been proposed for explaining the role of the IC in auditory fear conditioning (Casanova et al., 2016).

In support of this idea, previous work has shown that fear and anxiety responses may be modulated by afferent visceral signals. Rats with subdiaphragmatic vagal deafferentation showed reduced anxiety levels and attenuation of conditioned fear extinction (Klarer et al., 2014). Moreover, tone-elicited neural activity in the rat pIC was observed during both fear expression and the extinction of auditory fear conditioning (Casanova et al., 2018). It has recently been shown that a discrete population of neurons in the posterior IC responded to both electric footshocks and tone during threat learning and the 24-h memory test (Berret et al., 2019). Also, imaging studies have shown that changes in the cardiorespiratory state are represented in the insula (Hassanpour et al., 2017), fear processing is modulated by cardiac activity (Garfinkel et al., 2014), and human fear-related disorders such as anxiety are associated with altered interoceptive processing in the insular cortex (Paulus and Stein, 2010; Simmons et al., 2011).

Muscimol injected into the pIC prior to cat odor exposure impaired contextual threat conditioning. We observed a reduction in freezing when rats were re-exposed to the cat odor-paired context 24 h after muscimol injection. Numerous studies have demonstrated that learning occurs during exposure to cat odor cues. It has been shown that rats that returned to a place previously associated with predator cues displayed defensive behaviors and exhibited cardiovascular and neuronal changes (Dielenberg et al., 2001a; Staples et al., 2005). Studies have shown that muscimol completely abolished cortical electrical activity for approximately 9 h (Krupa et al., 1999). The most likely explanation of our findings is that the long-lasting effect of the muscimol inactivation of the pIC may have interfered with threat memory acquisition and/or consolidation processes (Casanova et al., 2016). However, there is evidence suggesting that drugs that are administered before or immediately after learning may induce state-dependent learning (Gisquet-Verrier et al., 2015; Osorio-Gómez et al., 2019). With the available dataset, the possibility that muscimol induced a state-dependent fear memory cannot be discarded because a critical control group (Mus-Mus) was not included. Notably, injection of neosaxitoxin into the pIC immediately after the first cat odor exposure caused a long-lasting reduction in freezing expression in subsequent exposures. Control animals exhibited significantly lower levels of freezing from the third exposure to cat odor onwards. This could be interpreted as a behavioral adaptation to cat odor as a result of repeated exposure to the stressor (i.e., habituation; Dielenberg and McGregor, 1999). Physiological responses to stressors also decline with rapid exposure to the same stressor. For instance, levels of corticosterone in the plasma increased after the first but not the fifth exposure to cat odor (File et al., 1993). Stress response habituation is correlated with a reduction in c-fos expression in several regions of the brain’s defensive system (Campeau et al., 2002; Weinberg et al., 2009). Our data suggests that, in addition to impaired threat-context association, the inactivation of the pIC might have facilitated stress response habituation. We have previously shown that neosaxitoxin infused into the hippocampus abolished neural activity for 48 h without inducing neural tissue damage (Galindo et al., 2018). We have also shown that intra-pIC neosaxitoxin injections did not impair motor functions (Casanova et al., 2016). The available data, however, does not permit us to discard the possibility of long-lasting effects of neosaxitoxin on IC functioning or neosaxitoxin-induced state-dependent memory.

Given that the IC is connected to the medial amygdala (Cádiz-Moretti et al., 2016), we speculate that the IC may exert its modulatory effect on the expression of innate defensive behavior acting through this subdivision of the amygdala which in turn is connected with the medial hypothalamic defensive circuit (Gross and Canteras, 2012). In support of this idea, it has been reported that the IC has a crucial role in modulating fear expression in auditory fear conditioning, acting through the lateral and central amygdala (Berret et al., 2019). It remains to be tested how, and through which projection pathways, the IC mediates innate defensive responses to cat odor.

The findings that muscimol-induced inactivation of the pIC reduced innate freezing expression and impaired contextual memory to predator odor appear to be compatible with a modulatory role of the IC in defensive responses. One recent report showed that the IC conveyed information about an aversive footshock to the amygdala, and has a prominent role in auditory threat learning (Berret et al., 2019). Moreover, our findings align with previous studies in rats showing that inactivation of the anterior IC immediately after training attenuated the behavioral and cardiovascular responses to the training context, supporting the involvement of the IC in the consolidation of contextual threat memory (Alves et al., 2013). We previously reported that inactivation of the pIC increased the latency to express gastric malaise and disrupted drug craving (Contreras et al., 2007) and that the anterior insula is involved in context/drug effect association (Contreras et al., 2012) as well as in consolidation of auditory fear memory (Casanova et al., 2016). The involvement of the IC in threat memory is also consistent with investigations showing that the IC plays an important role in taste memory formation (Bermúdez-Rattoni et al., 2004), object recognition memory (Bermudez-Rattoni et al., 2005), and social memory (Cavalcante et al., 2017). However, another limitation of the present study is that we cannot rule out the possibility that context pre-exposure before the first cat odor test could have decreased associative strength between cat odor and context cues (i.e., latent inhibition) which may have contributed to our findings.

Both the pIC and the anterior IC expressed higher levels of Fos-ir neurons after a single exposure to cat odor compared with the control collar. Freezing was highly correlated with the number of Fos-ir neurons in both the anterior IC and the pIC, which suggests that distinct neural populations within the insular networks may be involved in representing, and perhaps integrating, fear-related bodily states with olfactory predator cues. Interestingly, a second exposure to cat odor induced a different pattern of neuronal activity which could be explained by learning processes. We have recently reported that the population activity of neurons in the pIC was correlated with the expression of freezing behavior and that this pattern of activity changed during fear extinction, perhaps representing the extinction learning (Casanova et al., 2018).

Consistent with a previous report, we found that single cat odor exposure elicited a robust increase in Fos-ir neurons in both MeApv and VMHdm (Gross and Canteras, 2012), and Fos-immunoreactivity within these regions was correlated with freezing. A second cat odor exposure elicited a similar pattern, although with less activation in the VMHdm as shown previously (Staples et al., 2005). The reduced activation of the VMHdm during the second exposure could be due to a change in stimulus processing that occurs when the stimulus becomes familiar.

In conclusion, we found that the neural activity of the pIC is necessary for expressing innate freezing behavior and contextual threat learning to cat odor. These results provide further evidence that supports a role for the IC in fear expression and together with previous findings suggest that the IC may modulate fear responses, in particular, freezing, to both innate and learned environmental threats.

## Data Availability Statement

All datasets for this study are included in the article/Supplementary Material.

## Ethics Statement

The animal study was reviewed and approved by the institutional Bio Safety and Ethical Committee. Animal procedures were conducted in compliance with institutional guidelines by the National Institute of Health (USA) Guide for the Care and Use of Laboratory Animals (NIH Publication No. 80-23, revised 1996).

## Author Contributions

MR, MC and FT: conceived and designed the experiments. MR: performed the experiments. MR, MC, FC and PM: analysis of data. FT, MC and FC: contributed to reagents, materials and analysis tools. MC, MR and BH: wrote the article.

## Conflict of Interest

The authors declare that the research was conducted in the absence of any commercial or financial relationships that could be construed as a potential conflict of interest.
